# Notes and Comment

**Published:** 1892-03

**Authors:** 


### NOTES AND COMMENT.

The last annual report of the Postmaster-General presents an admirable showing in this important department of the public service. There is nothing nearer to the hearts of the people than its postal system, and the steady improvement of the letter service has been marked during the last twenty-five years. Not so, however, with reference to the newspaper and magazine branches. Here there is more incongruity of ruling and less harmony of action than in any section of the public service with which we are familiar. Some of these peculiarities are admirably set forth in a recent number of *Printers' Ink*, published by Geo. P. Rowell & Co., New York, and it would well repay all interested to carefully read what this admirable little paper says on the subject. One of the peculiarities of the service, which has always perplexed us to understand, is why a newspaper or magazine, for instance, can be sent from Buffalo to California for one cent a pound, whereas it costs two cents for every four ounces to deliver the same journal to a subscriber living in the city of its publication. In connection with the Postmaster-General's report it may be mentioned that he has published some admirable photographic reproductions of the late disaster on the Lake Shore Railway, whereby six postal clerks lost their lives. It is a graphic exhibition of the perilous nature of this service, and appeals to the sympathy of all mankind.

A dispatch from the Immigration Commissioner at New York assures Dr. Wende, the Health Commissioner, that none of the passengers who made the voyage on the "Massilia" were ticketed to or through Buffalo; hence it would appear that there is little danger of the typhus gaining foothold in the city from that source. However, it was a wise precaution that led Dr. Wende to appoint two competent physicians to inspect the incoming trains with reference to the possibility of their bringing into or through our city passengers who were infected with contagion. It is much easier to prevent than to cure, and no one will object to the strongest lines of preventive management, even though sometimes it seems to involve a useless expense. There is no waste of money when used in this manner, and we commend the prompt action of the Health Commissioner in this instance.

---

The *Texas Sanitarian* is a handsomely printed journal that appeared in November, 1891, as the advocate and champion of preventive medicine and hygiene. It is published at Austin, Texas, and is edited by Dr. T. J. Bennet, who seems to have an entire fitness for the position which he has assumed. There is no more important department of medical literature than that referring to sanitation and hygiene. We congratulate this new candidate for the high stand which it has taken, both as to the material which it presents and the method of its presentation.

---

The Western Society of Obstetricians and Gynecologists met for organization at Topeka, Kansas, December 29, 1891. Dr. M. B. Ward, of Topeka, was elected President, and the usual number of associate officers were chosen. This association is organized especially in the interest of men residing west of the Missouri river, for the investigation and study of the subjects covered by its title. The first meeting was a large and enthusiastic one, and we bespeak a successful career for the association.

---

The *Ohio Medical Journal*, which is the organ of the faculty of the Medical College of Ohio, appears on our table for the first time with its January number. It is a well-printed and good-looking organ, but has the disadvantage of being oversize. We wish that there could be some uniform standard of size adopted for medical journals. On the whole, the standard octavo seems to be the favorite, as it is best adapted to library shelves after binding.

THE *Hot Springs Medical Journal*, the first number of which was published January 15, 1892, comes to our table as an exchange. We hope it may be the means of improving the *esprit de corps* in the profession at that health resort.

---

THE *Albany Medical Annals* put on a new dress with the beginning of the year, and appears with a new editor at the helm. Dr. Howard Van Rensselaer will in the future conduct this able and progressive journal.

---

THE *Post-Graduate*, with its December issue, began a new series as a monthly. It is a progressive journal, and is always full of interesting reading matter.

